# Empowering PET: harnessing deep learning for improved clinical insight

**DOI:** 10.1186/s41747-023-00413-1

**Published:** 2024-02-07

**Authors:** Alessia Artesani, Alessandro Bruno, Fabrizia Gelardi, Arturo Chiti

**Affiliations:** 1 Department of Biomedical Sciences, Humanitas University, Via Rita Levi Montalcini 4, Milan, Pieve Emanuele 20090 Italy; 2 Department of Business, Law, Economics and Consumer Behaviour “Carlo A. Ricciardi”, IULM Libera Università Di Lingue E Comunicazione, Via P. Filargo 38, Milan, 20143 Italy; 3 Vita-Salute San Raffaele University, Via Olgettina 58, Milan, 20132 Italy; 4 Department of Nuclear Medicine, IRCCS Ospedale San Raffaele, Via Olgettina 60, Milan, 20132 Italy

**Keywords:** Artificial intelligence, Deep learning, Nuclear medicine, Positron emission tomography

## Abstract

This review aims to take a journey into the transformative impact of artificial intelligence (AI) on positron emission tomography (PET) imaging. To this scope, a broad overview of AI applications in the field of nuclear medicine and a thorough exploration of deep learning (DL) implementations in cancer diagnosis and therapy through PET imaging will be presented. We firstly describe the behind-the-scenes use of AI for image generation, including acquisition (event positioning, noise reduction though time-of-flight estimation and scatter correction), reconstruction (data-driven and model-driven approaches), restoration (supervised and unsupervised methods), and motion correction. Thereafter, we outline the integration of AI into clinical practice through the applications to segmentation, detection and classification, quantification, treatment planning, dosimetry, and radiomics/radiogenomics combined to tumour biological characteristics. Thus, this review seeks to showcase the overarching transformation of the field, ultimately leading to tangible improvements in patient treatment and response assessment. Finally, limitations and ethical considerations of the AI application to PET imaging and future directions of multimodal data mining in this discipline will be briefly discussed, including pressing challenges to the adoption of AI in molecular imaging such as the access to and interoperability of huge amount of data as well as the “black-box” problem, contributing to the ongoing dialogue on the transformative potential of AI in nuclear medicine.

**Relevance statement**

AI is rapidly revolutionising the world of medicine, including the fields of radiology and nuclear medicine. In the near future, AI will be used to support healthcare professionals. These advances will lead to improvements in diagnosis, in the assessment of response to treatment, in clinical decision making and in patient management.

**Key points**

• Applying AI has the potential to enhance the entire PET imaging pipeline.

• AI may support several clinical tasks in both PET diagnosis and prognosis.

• Interpreting the relationships between imaging and multiomics data will heavily rely on AI.

**Graphical Abstract:**

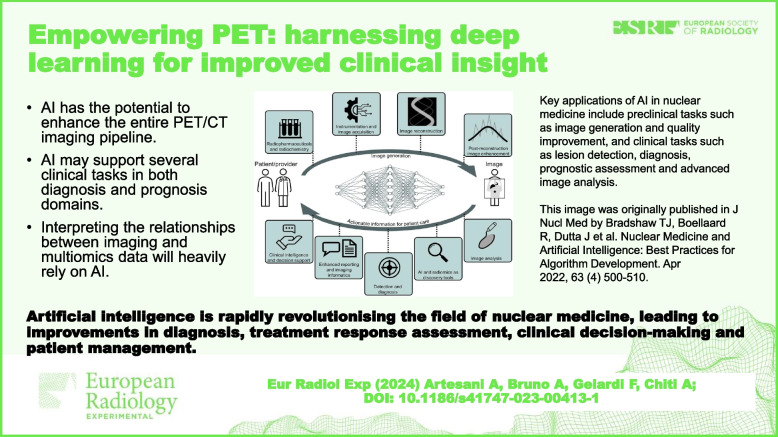

## Background

Over the past decade, there has been a significant progress in nuclear medicine imaging techniques, boosted by remarkable technological advances. Nuclear medicine physicians now have access to high-dimensional and multimodal images, along with a huge amount of quantitative data on the biological and genetic characteristics of tumours. In the era of precision medicine, the ability to improve image quality, harness this data, and extract meaningful information is a contemporary challenge in the oncological field. In this context, the implementation of artificial intelligence (AI) techniques is leading to significant breakthroughs, fundamentally transforming how clinicians approach medical diagnosis and patient care [1]. 

**Fig. 1:**
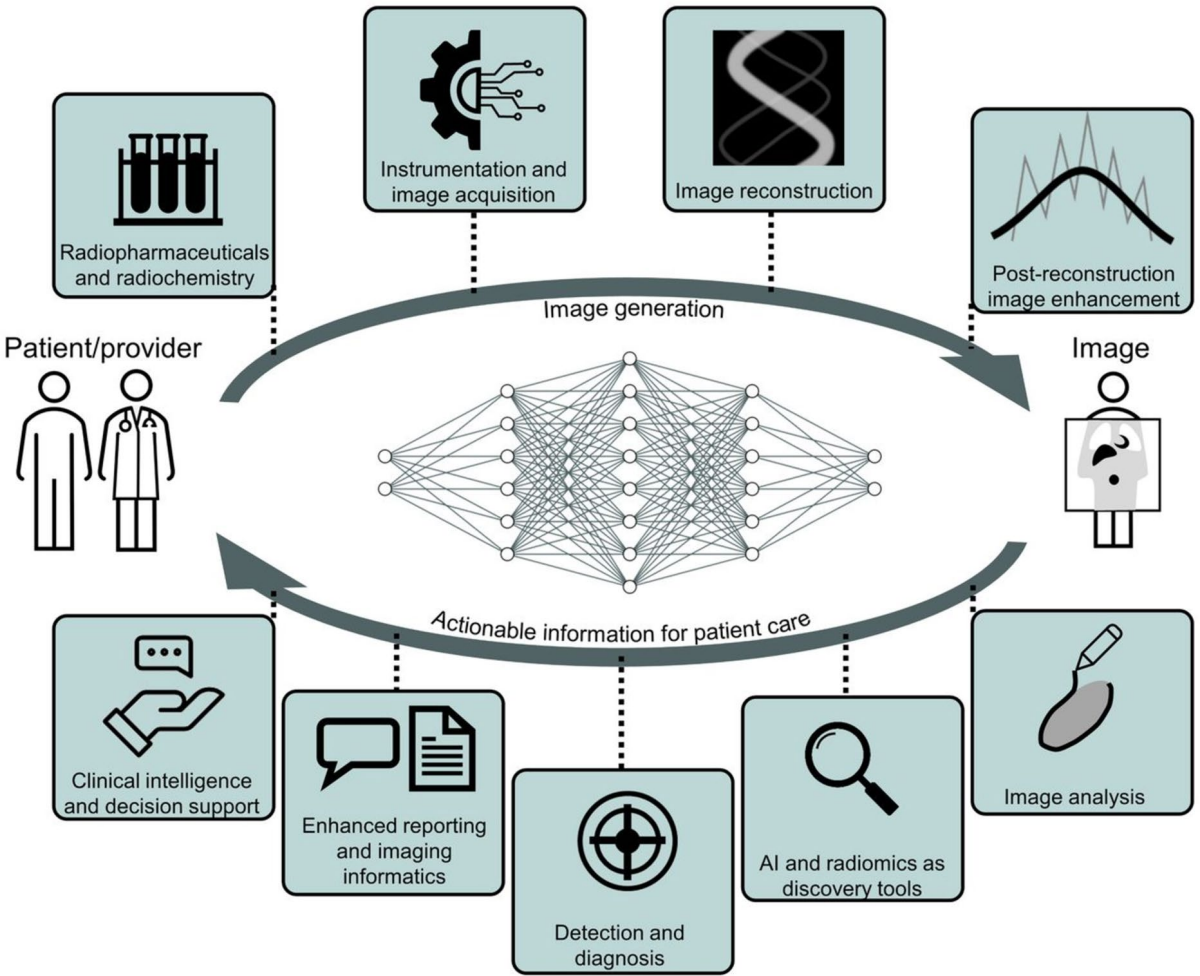
Key applications of artificial intelligence in nuclear medicine. Reprinted from JNM [5]

The integration of AI and positron emission tomography (PET) has clearly pushed the boundaries of these functional techniques. The growing synergy between AI and PET imaging promises to revolutionise diagnosis, improve accuracy and expand clinical utility. More than other AI techniques, deep learning (DL) has emerged as key player, finding applications in a wide range of tasks, that include imaging acquisition and quality improvement, as well as clinical activities (Fig. 1) [2]. Nevertheless, it is crucial to acknowledge that AI encompasses a broader spectrum of techniques beyond DL, such as classical machine learning, reinforcement learning, and hybrid AI models, albeit these are primarily niche applications within the field [3, 4].

This review will present some key AI applications within the PET imaging domain, including image generation, reconstruction, and restoration. A series of real-world clinical applications will be showcased, highlighting the tangible advantages that AI offers across various aspects of nuclear medicine, and its capability to reshape the field. Nonetheless, a critical discussion about the crucial issues related to data interoperability and AI black-box problem will be given, providing an overarching overview into both potential and limitations of AI within the field.

## Image generation development

### Image acquisition

The impact of AI algorithms begins in the PET scanner room, in the critical task of PET image acquisition, and includes event positioning, noise reduction through time-of-flight estimation and scatter correction. Optimising these three different aspects of PET image generation can significantly improve the overall image quality, with the tangible consequence of increased lesion detectability.

#### Event positioning

**Fig. 2:**
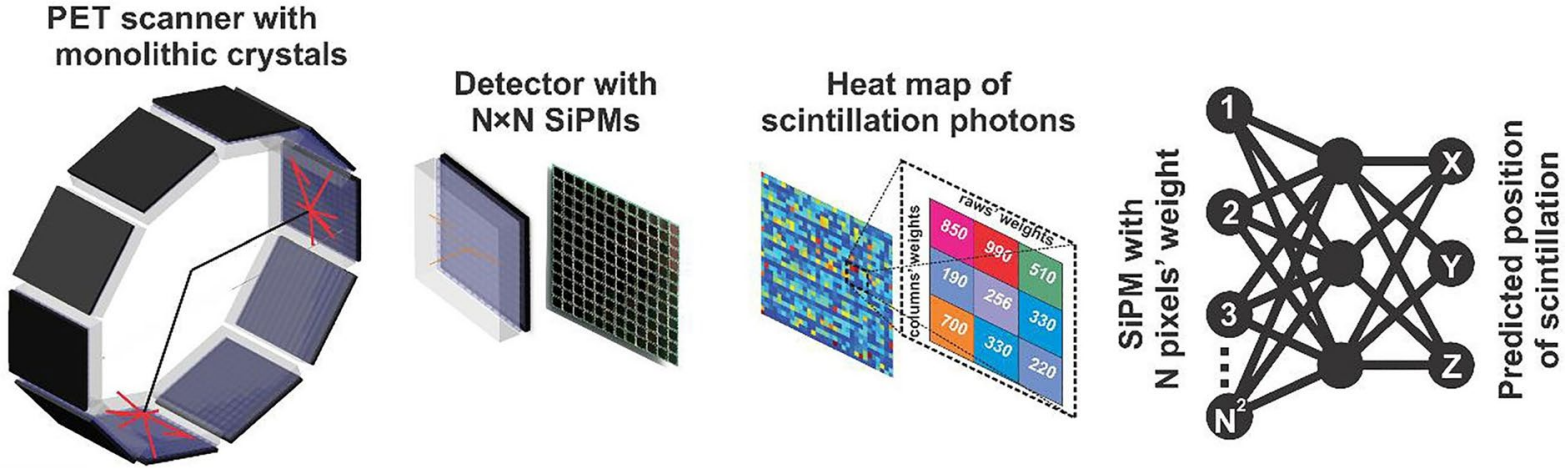
Deep learning-based event-positioning in positron emission tomography (PET) scanners with monolithic crystals. The three-dimensional coordinates of the scintillation position can be predicted by a multilayer perceptron neural network using the number of fired silicon photomultiplier (SiPMs) pixels and the total deposited energy. Reprinted with permission from Physica Medica [8]

Historically, simple signal processing methods have been used to compute the timing of pick-off for each detector waveform, using analogue pulse processing electronics and pixelated crystals [6]. With the advent of fast waveform digitisers, DL methods have become fundamental for improving the gamma detection from scintillators, predicting the time-of-flight (TOF) of photons, and improving the coincidence-time-resolution. AI-driven techniques have also been critical for the introduction into clinical applications of monolithic scintillator crystals, which are attracting the interest in the nuclear imaging community due to their reduced production costs, good spatial resolution, and depth-of-interaction estimation. The adoption of real-time algorithms to improve spatial resolution and make it comparable to pixelated crystals is a challenge for monolithic-based PET detectors. To solve complex non-linear tasks such as the determination of the photon interaction position, deep learning neural network algorithms have been fundamental for event time-stamping in monolithic crystals, drawing attention to the real-time hardware implementation essential for its use in a complete PET scanner (Fig. 2) [7]. Thanks to the implementation of deep learning neural network algorithms, monolithic-based devices now have potential to attain performance levels beyond the state-of-the-art [7].

#### Noise reduction through time of flight estimation

The ability to add information about the position of the positron annihilation along the Line-of-response (LOR) is an important parameter in the reconstruction process, leading to an improvement in signal-to-noise ratio and lesion detectability. Deep convolutional neural networks (CNN) have been implemented to estimate the TOF directly from the pair of digitised detector waveforms for a coincident event [6, 9]. CNNs have the ability to learn complex representations of the input data, making them suitable for TOF estimation from the waveforms that are confounded by multiple complex random processes [10]. Since all the timing information is contained in the first few nanoseconds of the detector waveforms, the CNN algorithms are ingeniously developed on the rising edge of the signals, without the need to store the entire waveform for TOF estimation. So far, simulation studies have shown that, for digitisers with monolithic PET detectors, a superior time resolution can be achieved with 3D-CNN, reaching an improvement of 26% compared to the traditional method of leading-edge discrimination followed by an averaging of the first few time-stamps [9].

#### Scatter correction

Another source of uncertainty affecting image quality is randomly scattered photons, which add low frequency backgrounds and introduce serious artefacts. AI methods do not completely replace traditional methods in this task, such as the physical model of photon scattering, but rather they represent an auxiliary means to find function-mapping relationships and largely depend on the model structure, data range, and training process [11]. Neural network (NN) approaches have been developed to solve the issue of the triple coincidence produced by photon scattering in LOR assessment [12]. The method computes the LOR within the coincidences by pre-processing the energy and position measurements, and then NN discrimination. The results with NN approaches showed very good LOR recovery rate (75%), yielding an overall high sensitivity increase of 55% (real scanner conditions) by incorporating triple coincidences within a traditional 360–660 keV energy window and a single energy threshold of 125 keV. When compared to photopeak-only images, the method demonstrated acceptable, limited resolution degradation with little to no contrast loss [12].

### Image reconstruction

PET scanners do not generate data directly in image space, but they require reconstruction algorithms to obtain a tomographic representation. This is an inverse problem that however lacks an exact solution, and only closed-form approximation can be found with iterative algorithms, which are computationally expensive and may still include modelling errors in the forward operator. DL-based approaches have been used to solve these limitations by replacing the uncertain user-defined variables in traditional methods with parameters learned directly from data.

Currently, there are two main AI approaches in PET image reconstruction [13]: data-driven and model-driven.

#### Data-driven approach

The NN learns to reconstruct the image directly from projection data, via a latent feature space, to decode to the desired image. Convolutional encoder–decoder networks are typically used because of their capability to compute image-to-image translation tasks. The overall mapping is usually trained by supervised learning for considering noise and deliver inference from the ground-truth object used in the training phase. In this case, the input raw PET data are three-dimensional sets of measured sinograms that are used to map the output three-dimensional images. At present, data-driven approaches are still impractical, as they require huge computational memory and training set size for small reconstructions. Moreover, they can be hardly generalised for unseen.

#### Model-driven approach

This is the most promising, and it is a physically informed approach. It integrates the existing state-of-the-art of statistical iterative image reconstruction methods into a deep network. DL is involved in the cascade successive reconstructions to provide rich, data-informed, prior information to the iterative process, making repeated use of the raw data. The model-driven approach has a highly reduced need for training data, since the physics and statistics of PET data acquisition do not need to be learned from scratch.

### Image restoration

The noise generated by the randomness of physical processes (annihilation) and scattering is one of the primary factors in PET image degradation, limiting the detectability of lesions and leading to inaccurate diagnosis. The same purpose of noise reduction is linked to the effort of dose reduction to limit the radiation exposure, which, however, results in an increase of image quality degradation. Noise suppression using iterative algorithms is not the best choice, as it might induce artefacts or poor-quality results. Instead, DL offers a highly advantageous approach and there are several solutions that use either supervised or unsupervised methods in image post-processing for denoising purposes [10].

#### Supervised method

**Fig. 3:**
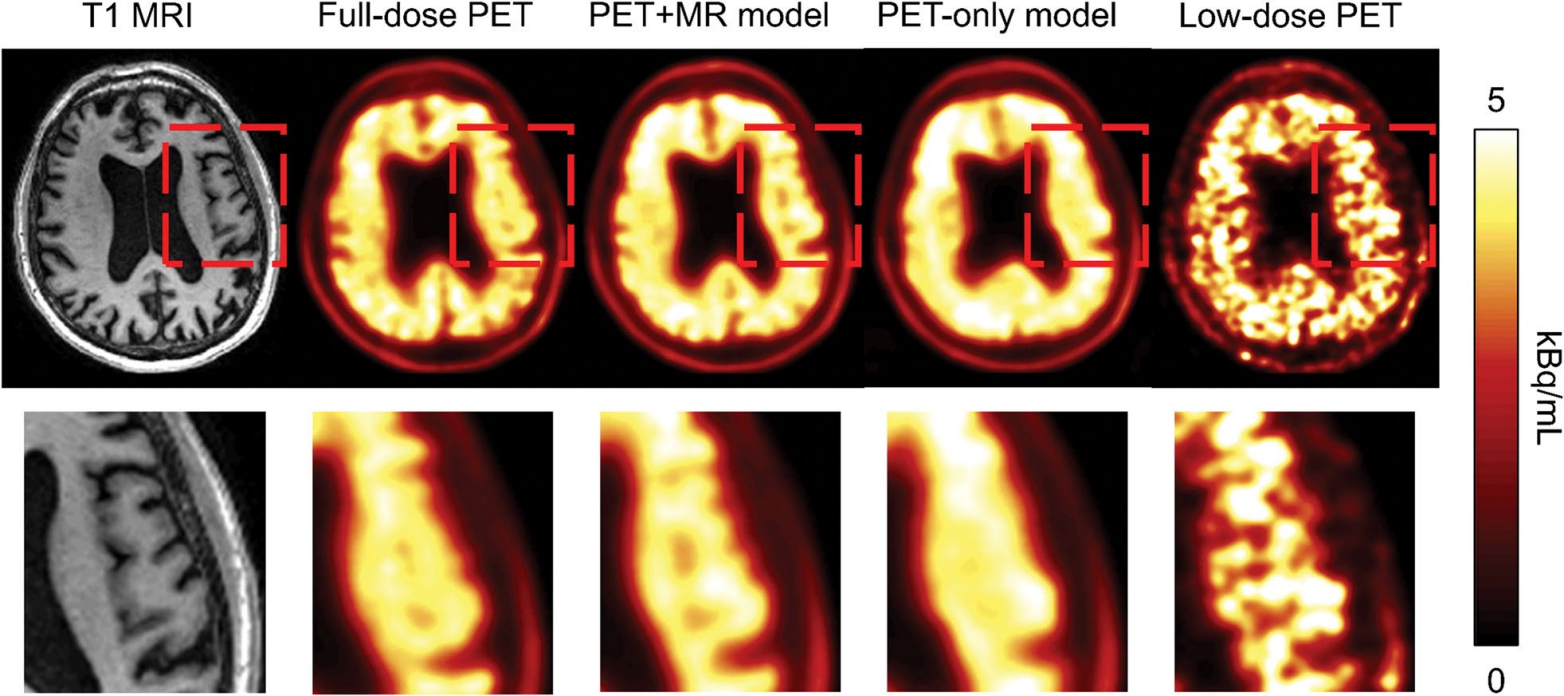
Example of image enhancement using a deep learning model of low-dose [^18^F]Florbetaben PET images of a patient with Alzheimer disease. The images demonstrate the superiority of including magnetic resonance imaging (MRI) data in the model over PET data alone. Reprinted with permission from Radiology [14]. *PET* Positron emission tomography

It can be performed by training a NN to map low and high-quality images, treating such a prediction as a regression problem. Both simulations and experimental data can be used as training targets, while the set of measured data can be obtained by introducing artificial noise prior to reconstruction. The relationships between the low-quality and high-quality images are then learned by the AI model, sometimes using anatomical priors from computed tomography (CT) or magnetic resonance imaging (MRI) as an additional network input channel to improve PET image quality. CNNs are used to predict full-dose PET images from PET/MRI or PET/CT images acquired at a quarter or less of a full dose (Fig. 3) [14, 15]. In a more recent study, Kaplan et al. [16] proposed a CNN method to further reduce the dose to one-tenth of the full dose of PET images by improving the preservation of edges and structural details of the network by including them in the loss function during training.

#### Unsupervised method

This approach is more commonly used to perform general tasks such as denoising, super-resolution and inpainting. A randomly initialised CNN can itself serve as a prior for image restoration by treating the low-quality images as training labels, and it has been shown that it is possible to stop training at a point where the network has learned the signal but not yet the noise [17]. Where possible, the random input can be replaced with a prior image containing additional information, such as the CT or MR image for hybrid PET/CT or PET/MRI denoising. Besides using DL methods for image restoration as a post-processing tool, it can also be incorporated into the iterative image reconstruction procedure as a replacement for traditional regularisation schemes [18]. With this approach, the network is trained to generate the image estimate at each updating step from a prior image and to perform a denoising step between each update, ensuring a higher data consistency on the final denoised image.

## Motion correction

Prolonged PET image acquisition times lead to unavoidable respiratory motion in organs and lesions, causing blurring that degrades image quality and reduces spatial resolution. Accurate assessing of areas affected by both respiratory and cardiac motion is challenging also due to the inherent limitations of PET spatial resolution. This is critical in clinical contexts such as detection of small lung nodules, quantification of myocardial blood flow and assessment of subtle changes in myocardial signal intensity (*i.e.*, suspected endocarditis or aortic root complications after vascular graft surgery) [19–22]. Data-driven gated approaches offer viable solution by modelling and compensating for these typical cardiac and respiratory motions, thereby enabling improved image reconstruction [23, 24].

Shi et al. [25] developed a DL-based automated motion correction for dynamic cardiac [^82^Rb]PET. They achieved superior performance in terms of motion estimation and myocardial blood flow quantification accuracy compared to conventional registration-based methods.

## Clinical applications

### Segmentation

Image pre-processing techniques using of regions of interest (ROI) segmentation are a critical step for several clinical tasks, including quantitative analysis, treatment planning, response assessment, lesion classification, and advanced image analysis (*i.e.*, radiomics). Several approaches have been developed to perform semi-automated, which still requires the manual adjustment of segmented regions, and fully-automated segmentation of ROI. AI will progressively replace the practice of manual segmentation, which is typically time-consuming, suffers from intra- and inter-reader variability, and low reproducibility. The most common segmentation algorithms include (1) threshold-based algorithms, which distinguish a fixed fraction or percentage of tracer uptake to define the target; (2) gradient-based algorithms, which recognise areas of high uptake from those of low uptake, enabling accurate delineation of inhomogeneous targets; (3) region-growing-based algorithms, which identify a seed region within the target and progressively include neighbouring voxels that meet certain similarity criteria; (4) algorithms based on statistical analysis; (5) AI-based through DL algorithms [26].

DL-based segmentation algorithms have been successfully applied to PET images with different radiopharmaceuticals and in different clinical settings, from oncology to cardiac and brain imaging [26–28]. Moreover, the full potential of hybrid imaging can be exploited by combining segmentation algorithms performed separately on CT images and PET images to improve the performance of segmentation models based on CT or PET images alone [29–31].

### Detection and classification

The most complex task delegated to AI in medical image analysis is the detection and classification of neoplastic lesions. A class of computer systems has been developed to assist physicians, known as computer-assisted detection and computer-assisted diagnosis systems. While the former merely identify and locate suspicious alterations, the latter also define their characteristics and classify them as benign/malignant findings. While these systems save time in image interpretation, they are not designed to replace the physician, whose expert eye is always required to confirm the result generated by the algorithm [32–34]. AI algorithms would provide pre-screened images and pre-identified key features, allowing for greater effectiveness and efficiency, by reducing the number of human errors, inter-observer variability and average reporting times. Computer-assisted systems can use either ML or DL algorithms, with supervised, semi-supervised and unsupervised approaches. The workflow includes a preprocessing phase to suppress unwanted noise, segmentation of a ROI, feature extraction and selection of meaningful characteristics, and lesion classification [32–35].

**Fig. 4:**
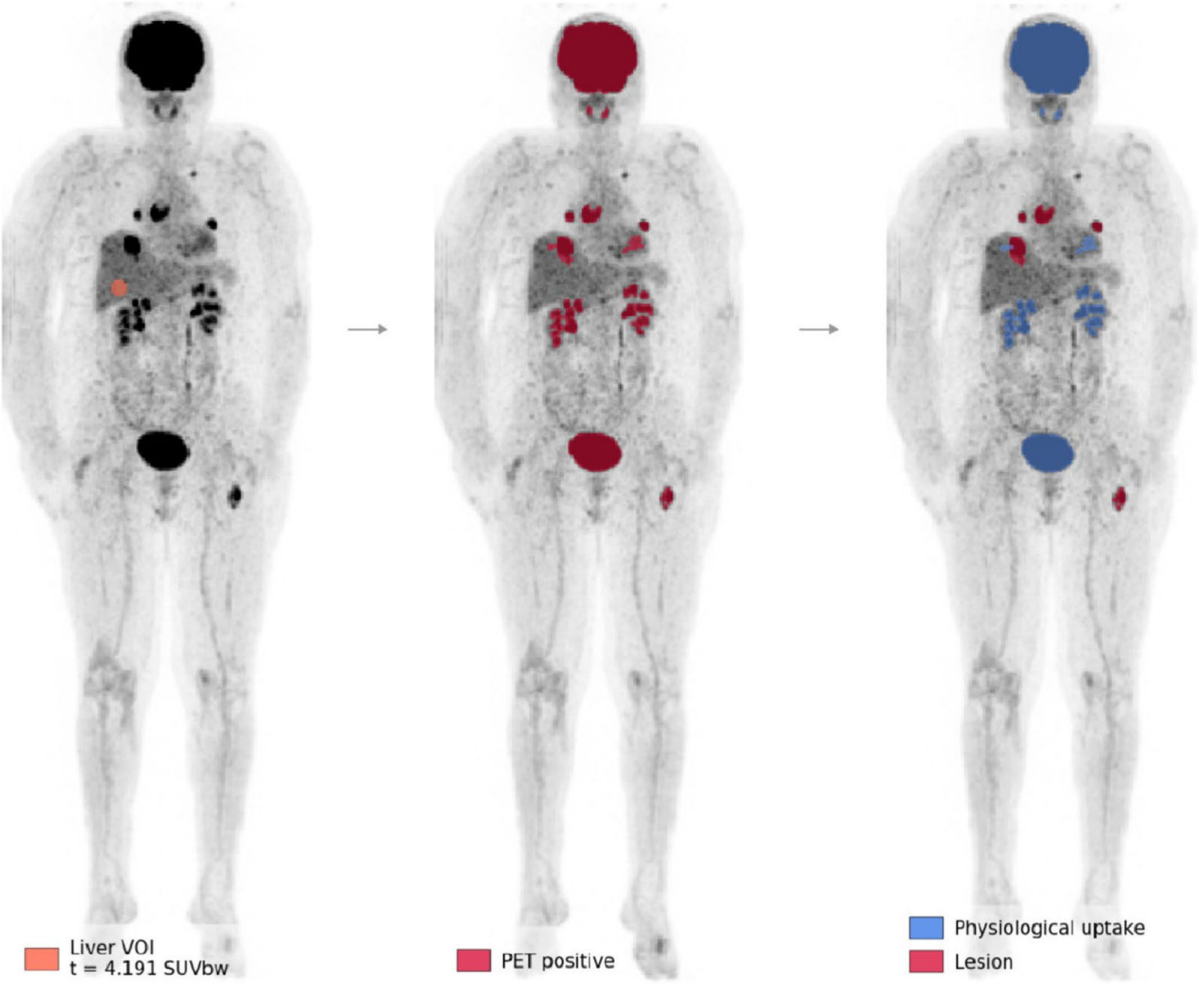
Illustrative demonstration of the results generated by a deep learning classification system on [^18^F]FDG PET/CT. This exhibit showcases the placement of the liver’s volume of interest (left), the segmentations following the application of a threshold (centre), and the categorisation into either physiological uptake or pathological lesions (right). Reprinted with permission from Eur J Nucl Med Mol Imaging [39]. *CT* Computed tomography, *FDG* Fluorodeoxyglucose, *PET* Positron emission tomography

Lung nodule characterisation is one of the most promising applications of computer-assisted systems. Several algorithms have been implemented with good performance rate on [^18^F]Fluorodeoxyglucose ([^18^F]FDG) PET/CT for the detection, classification and accurate staging of lung lesions [30, 31, 36–38]. A more complex task is the evaluation of whole-body PET/CT images, for instance in lymphoma and melanoma patients. CNNs have been used to correctly stage the disease by identifying different uptake patterns between suspicious and non-suspicious findings (*i.e.*, areas of increased physiological uptake) (Fig. 4) [38, 39]. Sibille et al. [38] developed a CNN to detect areas of increased uptake, to identify the anatomical location and to classify these areas as benign/malignant in patients with lung cancer and lymphoma. The CNN achieved high performance in terms of both anatomical localisation accuracy and discrimination of pathological lesions. Although the main field of application is oncology, AI-based classification systems have also been applied to various non-oncological molecular imaging techniques [8, 40]. Choi et al. applied DL to brain [^18^F]FDG PET for the diagnosis of neurodegenerative disorders. The DL model showed strong performance in discriminating patients with Alzheimer disease from normal controls. The model was also able to predict conversion to Alzheimer disease in patients with mild cognitive impairment and identify Parkinson disease patients with dementia [41].

### Quantification

Quantification is an essential biomarker for both diagnostic and therapeutic purposes. In oncology, the most commonly used semiquantitative parameters derived from [^18^F]FDG PET/CT reflect metabolism on a single voxel basis (“standardised uptake value”) or on a volumetric basis (“metabolic tumour volume” [MTV] and total lesion glycolysis). Semi-quantitative parameters provide relevant information for lesion characterisation, prognostic stratification, assessment of disease severity, and response to therapy, guiding clinicians in patient management. However, in certain conditions, many lesions must be segmented to obtain these data, and the task can be challenging and time-consuming. DL algorithms have been applied to whole-body images to segment multiple lesions and extract relevant semi-quantitative parameters quickly and automatically [42, 43]. In lymphoma, total MTV (*i.e.*, the sum of the MTV of all lesions) is a recognised parameter to stratify the risk of refractoriness/recurrence after first-line chemotherapy. Lymphoma is characterised by a high variability in the number, size, distribution, shape of lesions and metabolism. Several CNN models have been developed to segment pathological lesions, discard areas of physiological uptake and fully automatically calculate TMTV. These architectures may potentially provide clinicians with a high-throughput platform to perform semi-quantitative analyses efficiently and accurately [44–46].

Juarez-Orozco et al. applied DL to quantitative myocardial perfusion polar maps from [^13^N]NH_3_ PET to identify patients at risk of major adverse cardiac events. This approach significantly outperformed traditional clinical and functional variables, providing improved clinical prognostic estimates at the individual level [47].

### Treatment planning

Great technological advances prompted the development of advanced radiotherapy techniques which require a high degree of accuracy in defining the target tumour volume to minimise the radiation dose to surrounding healthy tissue and organs at risk, fitting perfectly into the modern vision of precision medicine. The delineation of the target volume (“gross tumour volume” [GTV]) is currently performed using a multimodal approach. In this perspective, metabolic data provide complementary information to morphological imaging, identifying the most aggressive tumour areas prone to radio-resistance mechanisms. The dose painting approach entails delivering a higher dose of radiation to metabolically active tumour areas, defined as “biological target volume”, for improved disease control and survival. In clinical practice, the delineation of GTV on PET/CT images is performed with semi-automatic threshold-based systems detecting tumour areas with high metabolism which are subsequently visually adjusted by the physician [48].

**Fig. 5:**
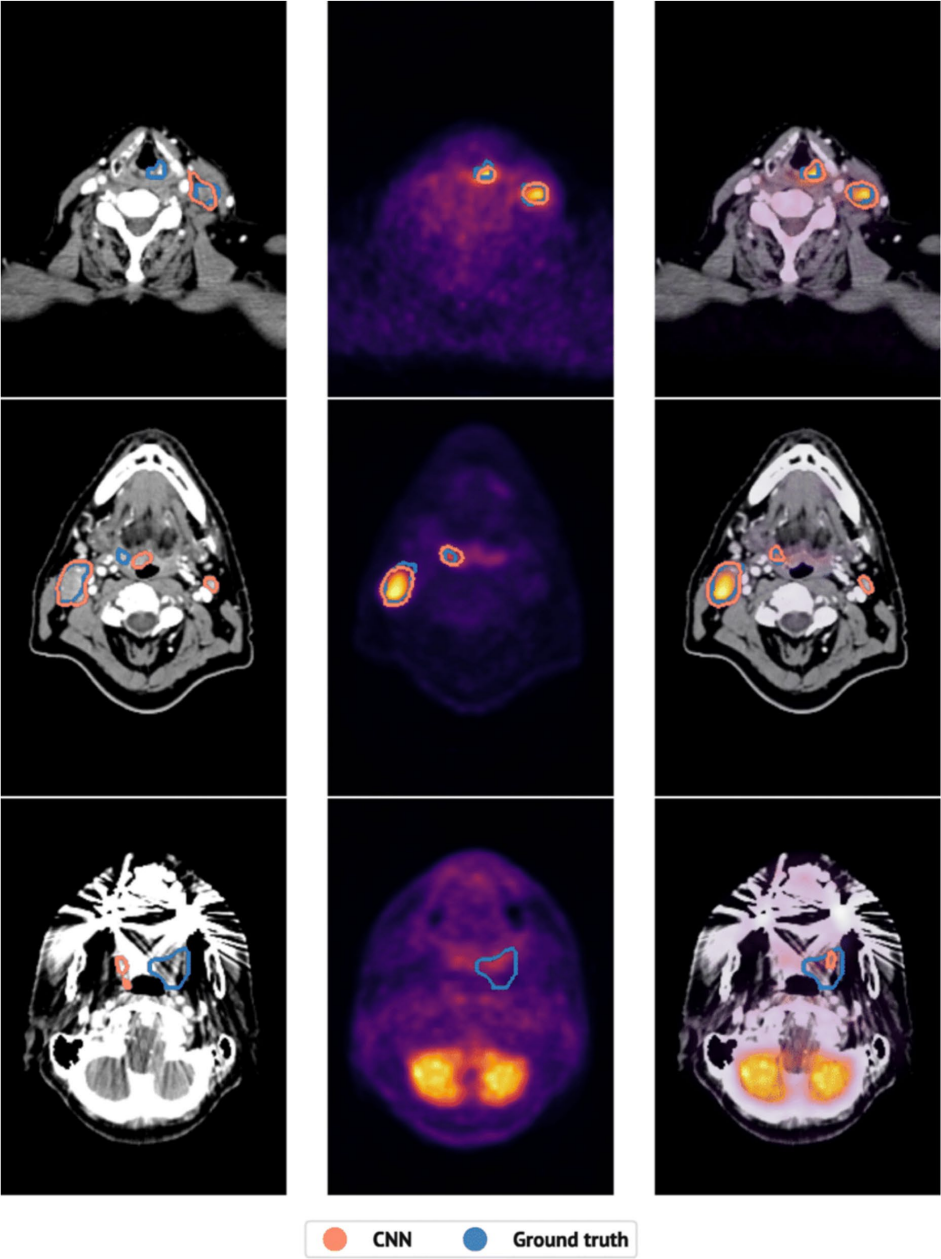
Application of CNNs for fully automated delineation of the GTV on [^18^F]FDG PET/CT in patients with head and neck cancer. The image shows the predicted (in red) and manual (in blue) segmentations in three different patients on CT alone, PET alone and PET/CT images (an experienced oncologist assigned qualitative scores of 10, 8, and 2 to the PET/CT-based predictions from top to bottom). Reprinted with permission from Eur J Nucl Med Mol Imaging [51]. *CNN* Convolutional neural networks, *CT* Computed tomography, *FDG* Fluorodeoxyglucose, *GTV* Gross tumour volume, *PET* Positron emission tomography

Deep CNN systems have also been applied to the automated delineation of different tumour histotypes to provide a simpler and faster procedure with less inter-observer variability. Several studies conducted in patients with head-neck cancer confirm a high degree of overlap between the biological target volume delineation of the primary tumour and pathological loco-regional lymph nodes proposed by CNN systems and that performed by expert physicians (Fig. 5) [49–51].

### Dosimetry

Predicting individual radiopharmaceutical dosimetry in compliance with the optimisation principle is crucial in therapeutic settings [52]. Currently, certain radioligand therapies are still administered with a fixed dose and pre-treatment imaging is used only to select candidates expressing the therapeutic target [53, 54]. However, a radical paradigm shift is taking place with the increasing development of radiopharmaceuticals for therapy. Whole-body absorbed dose quantification can be performed on planar or three-dimensional images employing different methods. The Medical Internal Radiation Dose Committee formalism is a simplified method for performing organ-level dosimetry. This approach assumes a uniform distribution of radiopharmaceutical activity and ignores different anatomical characteristics of the patient. The Monte Carlo simulation overcomes this inherent limitation but suffers from a high computational burden. Moreover, pre-therapy dose estimation involves several technical problems, requiring several dynamic whole-body scans for the extrapolation of pharmacokinetic data, which are currently not suitable for routine clinical practice [55, 56]. DL algorithms were used to generate individual voxel-based dosimetry maps from PET and CT with excellent results. The algorithms predicted absorbed doses with high precision, outperforming the Medical Internal Radiation Dose Committee and Monte Carlo methods both in terms of accuracy and computational efficiency [57, 58].

### Radiomics and radiogenomics

Over the past decade, there has been much debate about advanced image analysis using radiomics techniques and its applicability to clinical routines in the near future. Radiomics has rapidly attempted to establish relationship between visual image descriptors and biological/clinical endpoints. Radiomics involves the high-throughput extraction of high-dimensional quantitative features from medical images reflecting the biological characteristics of organs, tissues, and tumours, to increase the power of decision support models for outcome prediction and individualised patient management. The radiomics workflow includes several steps: image acquisition, ROI segmentation, feature extraction and analysis, database implementation with clinical and image-derived data and model building. Although the main application of radiomics is in oncology, the discipline is also expanding into non-oncological conditions. Saleem et al. [59] assessed the feasibility and utility of using textural features to diagnose aortoiliac graft infection from [^18^F]FDG PET/CT revealing promising results.

**Fig. 6:**
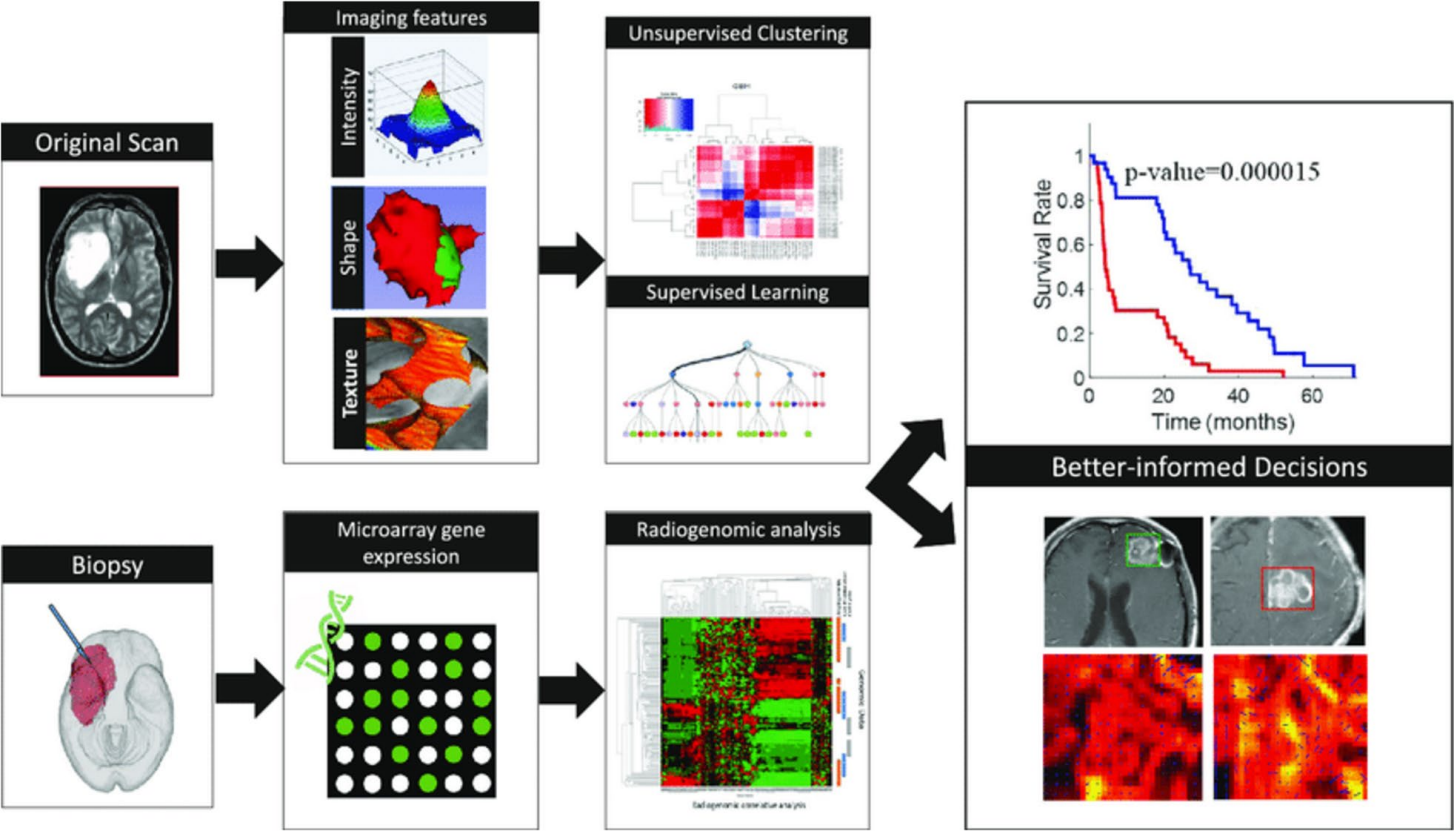
Radiomics and radiogenomics pipelines in outcome prediction. Reprinted from Br J Cancer [65]. The article is distributed under the terms of the Creative Commons CC BY license

Personalised medicine is an ambitious goal that relies on fusing multidisciplinary data from clinical, imaging, genomic, and other omic data [60]. Advances in genomics have led to the sequencing of entire genomes and have provided valuable insights into disease susceptibility and cancer development [61]. Radiogenomics is a relatively new scientific field that aims to discover new non-invasive biomarkers and to bridge the gaps between genomics and radiomics [62]. Recent trends include the use of AI and radiogenomics to support diagnosis, treatment decisions and prognosis in oncology, revolutionising healthcare [63]. The scientific literature provides several examples of AI-based radiogenomics methods for clinical applications, ranging from imaging features with genetic associations to tissue characterisation (Fig. 6) [64, 65]. Kirienko et al. [66] applied this approach to lung cancer patients. Excellent results were achieved in predicting tumour histology and patient outcome by combining radiomic analysis on [^18^F]FDG PET/CT and gene expression abnormalities via ML approach. Kim et al. [67]. explored both ML and DL to develop a prediction model for chemotherapy response and metastasis in paediatric patients with osteosarcoma using gene expression and image texture features from [^18^F]FDG PET/CT achieving high accuracy.

## Limitations and ethical considerations

While the integration of AI into nuclear medicine holds great promise, it is essential to recognise and address certain limitations and challenges. One of the foremost limitations to developing powerful and robust AI algorithms revolves around the quality and quantity of data available to train. The transition of AI-driven tools into clinical workflows can be hindered by limited data availability, accessibility, and interoperability. This is particularly true for rare cancers or specific patient populations, but also includes the variability in data quality between medical centres in terms of imaging protocols, equipment, and radiopharmaceuticals. Several strategies can help mitigate the limitations of insufficient data, including data augmentation, transfer learning, synthetic data generation, and one-shot learning [68–71]. Another approach is data sharing across different institutions. However, inadequate data interoperability can have a significant impact on the accuracy of AI predictions and a reference model should be embraced to establish robust data. Approaches to minimise measurement uncertainty, enable data identification, and standardise pre-existing data for research purposes are of paramount importance for data integration and enhanced AI applications in nuclear medicine [72]. Several open-source and proprietary software tools have been developed by exploiting the availability of large amounts of data for learning (open libraries, etc.). However, the integration of image interpretation software into clinical practice is limited by long development times and strict requirements for regulatory approval. Therefore, most algorithms are currently distributed as research tools. There is still a long way to go to provide evidence-based data and define the real impact of integrating AI into clinical practice [63, 73].

Another important limitation of AI in clinical practice is what is commonly referred to as the "black box" problem [74]. This refers to the inherent opacity of complex AI models, making their decision-making processes difficult to interpret and understand. In nuclear medicine, this challenge becomes particularly pertinent. Medical professionals cannot rely on the transparency and interpretability of results and instead must rely on AI-assisted diagnoses and treatment plans. This lack of transparency can potentially impede the widespread adoption of AI in clinical settings. Various approaches have been proposed to tackle this issue, such as incorporating retro-propagation of information from the model results back to the input data [75, 76]. In general, the development of techniques to improve the transparency and interpretability of AI models has been an ongoing effort. However, this remains an ongoing challenge within the discipline, demanding continued research and innovation to bridge the gap between the complexity of AI algorithms and the need for transparent and comprehensible decision-making processes in clinical practice.

## Future directions

The future of AI in PET imaging is bright, with several notable advances on the horizon. AI will play a key role in the development of accurate pseudo-CT methods for attenuation correction in PET/MRI. This will result in synthetic CT images from MR and PET data, improving PET quantification and anatomical precision while reducing radiation exposure [77]. A major trend, facilitated by AI-driven data fusion, is the integration of PET with other imaging modalities such as MRI and CT, as well as dynamic multiparametric PET analysis, providing a comprehensive view of a patient's condition, improved diagnostic capabilities and valuable clinical insights [78, 79]. Furthermore, AI will improve the feasibility of real-time imaging and intervention. Surgeons can use AI to navigate procedures and make critical decisions based on intraoperative PET imaging, resulting in greater surgical precision and minimised damage to healthy tissue [80].

## Conclusions

AI is rapidly revolutionising nuclear medicine, improving diagnostic accuracy, patient care, and the overall utility of PET imaging in both clinical practice and research. However, it is currently used mainly for research purposes and few applications have entered clinical practice. Responsible development and regulatory oversight will be essential to ensure that AI technologies are integrated safely and effectively into healthcare workflows.

## Data Availability

Not applicable.

## Abbreviations

AI: Artificial intelligence
CNN: Convolutional neural networks
CT: Computed tomography
DL: Deep learning
FDG: Fluorodeoxyglucose
GTV: Gross tumour volume
LOR: Line-of-response
MRI: Magnetic resonance imaging
MTV: Metabolic tumour volume
NN: Neural network
PET: Positron emission tomography
ROI: Region of interest
TOF: Time-of-flight
